# Eight years of experience with vismodegib for advanced and multiple basal cell carcinoma patients in the Netherlands: a retrospective cohort study

**DOI:** 10.1038/s41416-020-01220-w

**Published:** 2021-01-19

**Authors:** Babette J. A. Verkouteren, Marlies Wakkee, An K. L. Reyners, Patty Nelemans, Maureen J. B. Aarts, Emőke Rácz, Jorrit B. Terra, Lot A. Devriese, Robert-Jan Alers, Ellen Kapiteijn, Remco van Doorn, Marcel W. Bekkenk, Marie G.H.C. Reinders, Klara Mosterd

**Affiliations:** 1 Department of Dermatology, Maastricht University Medical Center, Maastricht, The Netherlands; 2 GROW School for Oncology and Developmental Biology, Maastricht University Medical Center, Maastricht, The Netherlands; 3 Department of Dermatology, Erasmus Medical Center Cancer Institute, Rotterdam, The Netherlands; 4 Department of Medical Oncology, University Medical Center Groningen, University of Groningen, Groningen, The Netherlands; 5 Department of Epidemiology, Maastricht University, Maastricht, The Netherlands; 6 Department of Medical Oncology, Maastricht University Medical Center, Maastricht, The Netherlands; 7 Department of Dermatology, University Medical Center Groningen, Groningen, The Netherlands; 8 Department of Dermatology, Isala Dermatologic Center, Zwolle, The Netherlands; 9 Department of Medical Oncology, University Medical Center Utrecht, Utrecht, The Netherlands; 10 Department of Gynecology, Maastricht University Medical Center, Maastricht, The Netherlands; 11 Department of Medical Oncology, Leiden University Medical Center, Leiden, The Netherlands; 12 Department of Dermatology, Leiden University Medical Center, Leiden, The Netherlands; 13 Department of Dermatology, Academic Medical Center, Amsterdam, The Netherlands

**Keywords:** Basal cell carcinoma, Metastasis

## Abstract

**Background:**

Vismodegib has been used for the treatment of locally advanced basal cell carcinoma (laBCC) and metastatic BCC (mBCC) since 2011. Most efficacy and safety data are provided by clinical trials. This study evaluates the effectiveness of vismodegib for the treatment of laBCC, mBCC and basal cell nevus syndrome (BCNS) patients, and the tumour characteristics associated with a higher probability of achieving a complete response in the Netherlands.

**Methods:**

A retrospective cohort study that included all patients ≥18 years with histologically proven basal cell carcinoma that received ≥1 dose of vismodegib between July 2011 and September 2019 in the Netherlands.

**Results:**

In total, 48 laBCC, 11 mBCC and 19 BCNS patients were included. Median progression-free survival was 10.3 months (95% confidence interval (CI), 7.5–22.6) for laBCC, 11.7 (95% CI, 5.2–17.5) for mBCC and 19.1 (95% CI, 7.4–20.2) for BCNS. Larger laBCCs were associated with a lower probability of complete response (hazard ratio (HR) 0.77 per increase in cm, *p* = 0.02). Of all BCNS patients, 63% received ≥2 treatment sequences with vismodegib; all achieved partial responses.

**Conclusions:**

Half of the aBCC patients progress within 1 year after the start of vismodegib treatment. More research is needed to investigate other treatment strategies after vismodegib progression and to evaluate long-term effects of repetitive vismodegib treatment.

## Background

Basal cell carcinoma (BCC) is the most common skin cancer worldwide.^1^ Therapeutic options vary from non-invasive therapies to local radiotherapy and surgery.^2^ However, if a BCC stays untreated, it can develop into an advanced BCC (aBCC), comprising locally advanced BCC (laBCC) and metastatic BCC (mBCC). Surgery or radiotherapy is not always an option for the treatment of aBCCs. In 2012, the Phase 2 ERIVANCE BCC trial investigated the efficacy and safety of vismodegib, the first-in-class molecule for targeted therapy for aBCCs that are not suitable for surgery and/or radiotherapy.^3^ Vismodegib inhibits the oncogenic protein smoothened (SMO), a downstream signal of the hedgehog pathway that plays an important role in the pathogenesis of BCC. Mutations in the hedgehog pathway are found in the majority of BCCs.^4^ An efficacy analysis of 96 patients in the ERIVANCE trial showed a median investigator-assessed progression-free survival (PFS) of 9.3 months (95% confidence interval (CI), 7.4–16.6) for those with mBCC and 12.9 months (95% CI, 10.2–28.0) for those with laBCC.^3^ Based on the results of this trial and under priority review as a first-in-class molecule, targeted therapy with vismodegib was registered for the treatment of laBCC and mBCC in the Netherlands.^5^ Another large Phase 2 trial assessed the safety of vismodegib (SafeTy Events in VIsmodEgib, STEVIE). The efficacy analysis of that trial included 1192 patients and showed a median investigator-assessed PFS of 13.1 months (95% CI, 12.0–17.7) for those with mBCC and 23.2 months (95% CI, 21.4–26.0) for those with laBCC.^6^ Of all patients, 98% experienced at least one adverse event, with the most frequently observed adverse events being muscle spasms, alopecia, dysgeusia, decreased appetite, decreased weight, and asthenia.^6^ In both the ERIVANCE BCC and STEVIE trials, only dose interruption of 4–8 weeks was accepted to recover from toxic effects and different treatment schedules were not allowed.^3,7^ Some patients need long-term treatment with vismodegib and an intermittent treatment schedule could possibly optimise the balance between benefit and side effects. This seems especially relevant in patients with basal cell nevus syndrome (BCNS), as BCCs will keep on developing in these patients during their entire lives. Therefore, the multiple basal cell carcinomas (MIKIE) trial compared two different intermittent dosing regimens for vismodegib in patients with either BCNS or high-frequency BCC (HF-BCC) patients.^8^ Both schedules showed similar response rates and adverse events rates; however, intermittent dosing was associated with fewer grade ≥3 treatment-emergent adverse events (TEAEs) compared to the STEVIE trial.^8^ The median duration of treatments in the MIKIE trial was 71.4 and 68.4 weeks depending on the dosing schedule, compared to 36.4 weeks for laBCC patients and 52.0 weeks for mBCC patients treated with the regular dosing schedule of 150 mg daily in the STEVIE trial.^7,8^ Unfortunately, extensive information about the indication, use, safety and (predictors of) effectiveness of vismodegib is still sparse.^9^ This study presents effectiveness, safety and the treatment course of all patients with aBCC or multiple BCCs who were treated with vismodegib in the Netherlands between July 2011 to September 2019.

## Methods

### Study design and patients

This retrospective, multicentre, longitudinal cohort study included all patients treated with vismodegib for aBCC or multiple BCCs in the Netherlands from July 2011 till 9 September 2019. In the Netherlands, vismodegib is only prescribed in seven academic medical hospitals (verified by contacting insurance companies), and all patients were gathered from these centres. All patients were aged ≥18 years, had a histologically proven BCC and received at least one dose of vismodegib. All indications for vismodegib treatment in BCC were included; laBCC, mBCC, multiple BCCs in BCNS and in non-BCNS patients. Vismodegib was either started in a clinical trial setting (STEVIE, *n* = 21 times, or MIKIE, *n* = 8 times) or in daily practice (*n* = 92 times).^7,8^ A new treatment sequence was defined as restarting vismodegib after a break of at least 8 weeks. Under the supervision of a dermato-oncologist (K.M.), two investigators, B.J.A.V and R.-J.A., extracted data from the electronic patient files and entered it into a standardised Castor database. This study was approved with a waiver of informed consent by the Medical Ethics Committee of all participating centres.

### Outcome measures

For the analysis on the effectiveness of vismodegib, the primary endpoint was the median PFS after the start of the first vismodegib prescription. Secondary endpoints were the difference in median PFS between the clinical trial and daily practice patients, probability of response (partial and complete) and PFS at 1, 3, 6 and 12 months, median duration of (complete) response and median time to all response endpoints (the period after which 50% of patients had reached the endpoint of interest). Response and progression were measured according to investigator-assessed clinical response as noted in the patient file. For the indication of multiple BCCs in (non-)BCNS patients, progression was defined as the development of new or recurrent BCCs. An additional analysis was performed to evaluate which patient and tumour characteristics were associated with an increased probability for achieving a complete response in the first treatment sequence. For this purpose, data were recorded on the duration of tumour presence, tumour size, histologic subtype, bone invasion, and previous therapy. Tumour measurement information was gathered from patient files, clinical photographs of the tumour and/or computed tomography or magnetic resonance imaging. Safety analysis included frequency, severity (measured according to the National Cancer Institute Common Terminology Criteria for Adverse Events, version 4.0) and reversibility of TEAEs.

### Data analysis and statistical method

Categorical variables were presented as percentages with absolute numbers and continuous variables as median with range, as appropriate. Time-to-event (Kaplan–Meier) analyses were used to estimate the cumulative probability of an endpoint at pre-specified follow-up periods as well as median time to endpoints. The observation period of patients started at the date of first treatment with vismodegib and ended at the date of first documentation of response or progression or at the date of death, depending on the studied outcome. A log-rank test was used to calculate differences between clinical trial and daily practice patients. For the median duration of response, the observation period started at the date of first documentation of response and ended at the date of first documentation of progression. For the patients who had not experienced the event of interest, observations were censored at the date of the last tumour assessment. Effectiveness analyses were performed on the first treatment sequence data. To evaluate characteristics associated with increased probability for achieving the complete response in the first sequence, univariable Cox regression analyses were performed and hazard ratios with 95% confidence intervals and *P* values were calculated. The variables with a significant or strong association (defined as at least halving of doubling of the hazard ratio) were entered into a multivariable Cox regression analysis to evaluate the independent effect of these variables. *P* values <0.05 were considered to indicate the statistical significance. Statistical analyses were performed with IBM SPSS Statistics version 25 and STATA version 13.0.

## Results

**Fig. 1:**
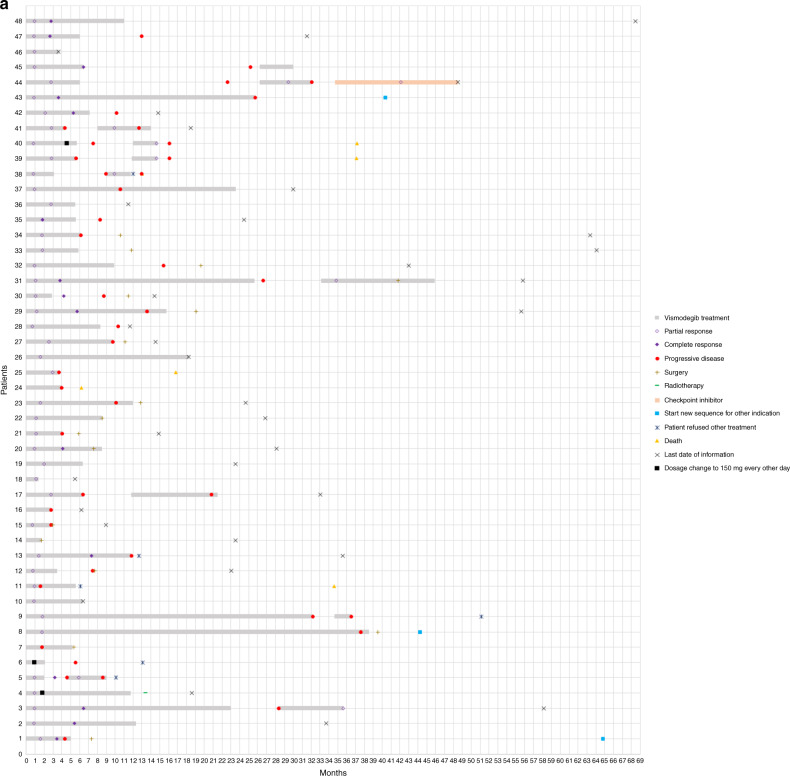

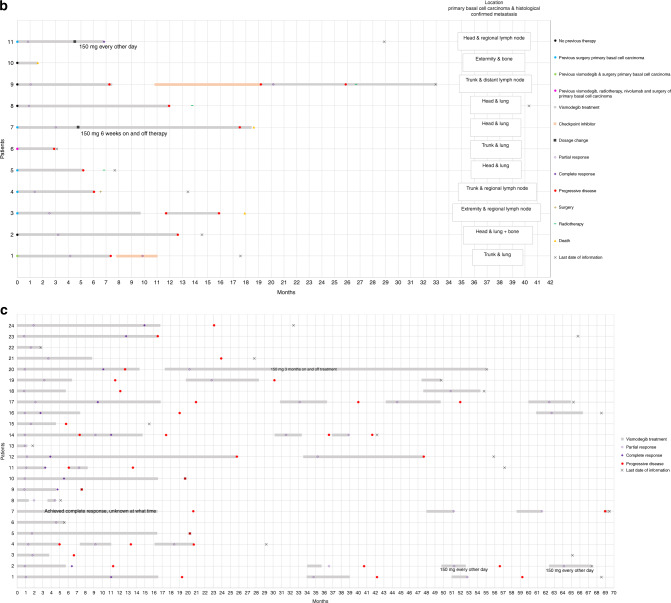
Swimmer plots of all individual patients per treatment indication. Time is shown on the horizontal axes in months and individual patients are shown on the vertical axes. **a** Swimmer plot of locally advanced basal cell carcinoma patients. Patients 14, 21, 22 and 23 received vismodegib as neoadjuvant therapy. Patients 8, 20, 28, 31 and 43 are basal cell nevus syndrome patients. **b** Swimmer plot of metastatic basal cell carcinoma patients. **c** Swimmer plot of multiple basal cell carcinoma patients. Patients 20 and 21 are high-frequency basal cell carcinoma patients; patients 22, 23 and 24 are xeroderma pigmentosum patients.

**Fig. 2:**
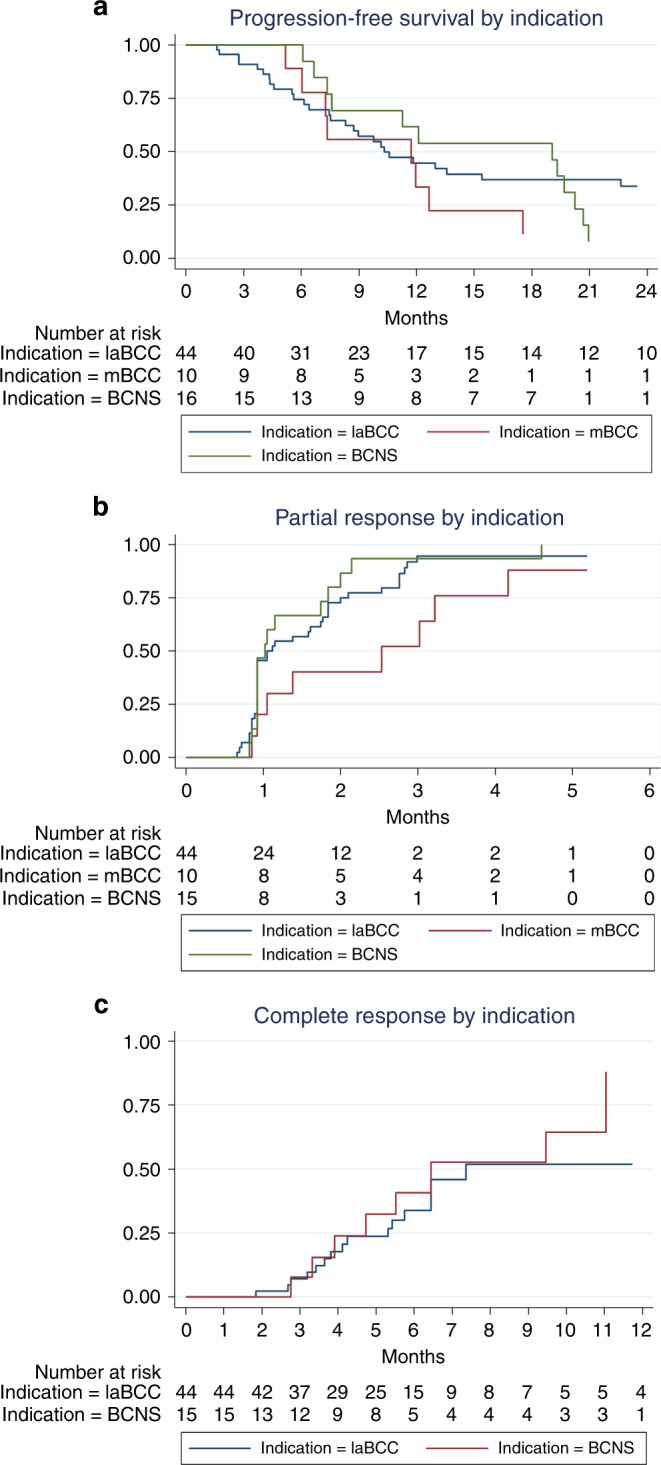
Kaplan-Meier curved of time-to-event analyses per treatment indication. (**a**) Progression-free survival by indication. (**b**) Partial response by indication. (**c**) Complete response by indication.

Between July 2011 and September 2019, 80 patients were treated with vismodegib in seven centres in the Netherlands. Patient, tumour and treatment characteristics can be found in Table 1. Fifty-one patients were treated with only one sequence, 21 with only two, 3 with three, and 5 with four. Swimmer lane plots per treatment indication can be seen in Fig. 1 and Kaplan–Meier curves from time-to-event analyses in Fig. 2 and Table 2.

**Table 1:** Patient, tumour and treatment characteristics.

	laBCC, *n* = 48	mBCC, *n* = 11	BCNS, *n* = 19	Multiple non-BCNS BCCs, *n* = 5
Sex				
Men, *n* (%)	24 (50%)	6 (55%)	12 (63%)	3 (60%)
Women, *n* (%)	24 (50%)	5 (45%)	7 (37%)	2 (40%)
Age at the start, median (range), years	75.5 (36–98)	70 (52–81)	46 (35–71)	77 (44-82)
<65 years	11 (23%)	4 (36%)	18 (95%)	1 (20%)
≥65 years	37 (77%)	7 (64%)	1 (5%)	4 (80%)
Caucasian, *n* (%)	48 (100%)	11 (100%)	19 (100%)	5 (100%)
Self-reported presence of BCC				
Median (range), years	6 (0.3–20)	5 (0.3–22)	–	–
Unknown, *n* (%)	14 (29%)	3 (27%)		
Basal cell nevus syndrome				
Yes, *n* (%)	5 (10%)	0 (0%)	19 (100%)	0 (0%)
No, *n* (%)	43 (90%)	11 (100%)	0 (0%)	5 (100%)
Previous treatment^a^				
None	20 (42%)	4 (36%)	0 (0%)	2 (40%)
Surgery	21 (44%)	6 (55%)	19 (100%)	5 (100%)
Radiotherapy	7 (15%)	1 (9%)	1 (5%)	2 (40%)
Cryotherapy	2 (4%)	0 (0%)	3 (16%)	2 (40%)
Curettage	1 (2%)	0 (0%)	3 (16%)	0 (0%)
Photodynamic therapy	2 (4%)	0 (0%)	4 (21%)	2 (40%)
5-Fluorouracil cream	2 (4%)	0 (0%)	3 (16%)	1 (20%)
Imiquimod cream	1 (2%)	0 (0%)	6 (32%)	0 (0%)
Laser (type unknown)	0 (0%)	0 (0%)	2 (11%)	0 (0%)
Other	2 (4%)	1 (9%)	2 (11%)	0 (0%)
Site laBCC				
Head and neck	40 (83%)	5 (46%)	–	–
Trunk	7 (15%)	4 (36%)	–	–
Extremities	1 (2%)	2 (18%)	–	–
Multiple sites	–	–	19 (100%)	5 (100%)
Size laBCC				
Median (range) (cm)	5 (1–30)	14.5 (4–22)	–	–
Unknown, *n* (%)	9 (19%)	5 (45%)	–	–
Subtype laBCC				
Infiltrative	37 (77%)	7 (64%)	–	–
Nodular	9 (19%)	0 (0%)	–	–
Unknown	2 (4%)	4 (36%)	–	–
Bone invasion laBCC				
Present, *n* (%)	16 (33%)	6 (55%)	–	–
Absent, *n* (%)	32 (67%)	5 (45%)	–	–
Site of metastasis				
Regional lymph nodes	–	3 (27%)	–	–
Distant lymph nodes	–	1 (9%)	–	–
Lungs	–	6 (55%)	–	–
Bones		2 (18%)	–	–
Duration of first treatment sequence				
Median (range), months	6.4 (1.4–38.5)	7.5 (1.6–18.5)	6.6 (1.2–25.7)	14.4 (2.8–16.8)
Start dosage				
150 mg daily	33 (69%)	11 (100%)	8 (42%)	2 (40%)
STEVIE	15 (31%)	0	6 (32%)	0
MIKIE	0	0	5 (26%)	3 (60%)
Short treatment interruptions				
Yes, *n* (%)	6 (12%)	0	1 (5%)	0
No, *n* (%)	42 (88%)	11 (100%)	18 (95%)	5 (100%)
Dosage change				
Yes, *n* (%)	3 (6%)	2 (18%)	1 (5%)	0
No, *n* (%)	45 (94%)	9 (82%)	18 (95%)	5 (100%)
Sequences^b^				
One	37 (77%)	9 (82%)	7 (37%)	4 (80%)
Two	11 (23%)	2 (18%)	5 (26%)	1 (20%)
Three	0	0	4 (21%)	0
Four	0	0	3 (16%)	0
Median duration between sequences, months (range)	6.0 (2.5–20.7)	6.9 (2.0–11.8)	11.2 (2.2–54.2)	3.0 (–)
Clinical review frequency in first sequence				
Monthly	37 (77%)	8 (73%)	19 (100%)	5 (100%)
2-monthly	9 (19%)	2 (18%)	0	0
3-monthly	2 (4%)	1 (9%)	0	0
Still on treatment				
Yes, *n* (%)	2 (4%)	1 (9%)	2 (11%)	1 (20%)
No, *n* (%)	46 (96%)	10 (91%)	17 (89%)	4 (80%)
Stop reason				
Tumour progression	15 (33%)	6 (60%)	1 (6%)	0
Toxicity	22 (48%)	2 (20%)	13 (76%)	2 (50%)
Vismodegib as neoadjuvans	4 (9%)	1 (10%)	0	0
Patient died	0	1 (10%)	0	0
No therapy compliance	2 (4%)	0	0	0
Physician fears development of resistance	2 (4%)	0	0^c^	0
End of trial	1 (2%)	0	3 (18%)	2 (50%)
Median duration of follow-up from the start of vismodegib treatment, months (range)	24.6 (1.8–83.4)	15.2 (1.6–40.3)	54.7 (1.8–68.5)	32.4 (2.8–65.8)

*laBCC* locally advanced basal cell carcinoma, *mBCC* metastatic basal cell carcinoma, *BCNS* basal cell nevus syndrome, *BCC* basal cell carcinoma.

^a^Percentages can add up to >100% because a patient can have had various previous treatments.

^b^For the specific indication and which was started in the Netherlands.

^c^Six following sequences were ended because the physician feared development of resistance.

**Table 2:** Time-to-event anayses of progression and response endpoints.

Indication/endpoint	1 month (95% CI)	3 months (95% CI)	6 months (95% CI)	12 months (95% CI)	Median time to (95% CI)	Median duration of response (95% CI)^a^
laBCC						
PFS overall	100.0	90.9 (77.6–96.5)	74.5 (58.6–85.0)	44.6 (29.1–58.9)	10.3 (7.5–22.6)	NA
PFS STEVIE	100.0	93.3 (61.3–99.0)	86.7 (56.4–96.5)	60.0 (31.8–79.7)	13.6 (6.1–26.6)	NA
PFS daily practice	100.0	89.7 (71.3–96.5)	67.8 (47.1–81.8)	35.4 (17.8–53.6)	10.2 (5.6–22.6)	NA
Partial response	45.5 (32.2–61.2)	94.6 (84.4–99.0)	NR	NR	1.1 (0.9–1.8)	9.7 (6.7–19.9)^b^
Complete response	0.0	7.1 (2.3–20.4)	33.9 (20.6–52.5)	51.9 (33.2–73.5)	7.4 (5.8–NE)	10.3 (4.5–22.1)
mBCC						
PFS	100.0	100.0	88.9 (43.3–98.4)	33.3 (7.8–62.3)	11.7 (5.2–17.5)	NA
Partial response	20.0 (5.4–59.1)	52.0 (25.5–83.9)	NR	NR	2.5 (0.9–4.2)	9.2 (3.2–14.5)^b^
Complete response	NA	NA	NA	NA	NA	NA
BCNS						
PFS	100.0	100.0	100.0	61.5 (30.8–81.8)	19.1 (7.4–20.2)	NA
Partial response	46.7 (25.6–73.7)	93.3 (74.0–99.6)	NR	NR	1.0 (0.9–1.7)	11.3 (5.0–18.8)^b^
Complete response	0.0	7.7 (1.1–43.4)	40.8 (19.3–72.2)	88.2 (59.8–99.3)	6.4 (3.9–11.0)	8.3 (2.8–16.3)

Cumulative probability of PFS, partial response and complete response with 95% CI, median time to endpoint with 95% CI and median duration of any and complete response with 95% CI.

*PFS* progression-free survival, *95% CI* 95% confidence interval, *NA* not applicable, *NR* no more responders, *NE* not estimable.

^a^Analysis based on responders only.

^b^Median duration of any response.

### LaBCC

A total of 48 patients received vismodegib for a laBCC, five of them had BCNS. Tumours were located in the head and neck region in 83% (*n* = 40), on the trunk in 15% (*n* = 7) and on the extremities in 2% (*n* = 1). Median self-reported tumour presence was 6 years (range, 0.3–20 years) and size was 5.0 cm (range, 1.0–30.0 cm), respectively. Thirty-seven tumours had an infiltrative component in the histologic sample (77%), nine were nodular (19%), and in two tumours (4%) this information was missing. Bone invasion was present in 16 of 48 tumours (33%). Of all 48 patients, 28 (58%) received at least one previous treatment for their tumour, mostly surgery or radiotherapy. At the start of vismodegib treatment, the median age of the patients was 75.5 years (range, 36–98 years).

#### Effectiveness

Four patients received vismodegib intentionally as neoadjuvant therapy and were therefore excluded leaving 44 patients for analysis. Median PFS was 10.3 months (95% CI, 7.5–22.6) for all 44 laBCC patients. There was no statistically significant difference in median PFS between daily practice and STEVIE trial patients (10.2 months (95% CI, 5.6–22.6) and 13.6 months (95% CI, 6.1–26.6), respectively (*p* = 0.39)). At 3 months after the start of vismodegib, the probability of partial response was 94.6% (95% CI, 84.4–99.0) and probability of complete response after 6 months of treatment was 33.9% (95% CI, 20.6–52.5), with a median duration of complete response of 10.3 months (95% CI, 4.5–22.1).

The HRs from the multivariable analysis showed a significantly decreased probability of achieving a complete response in larger tumours (HR 0.77 per increase in cm, *p* = 0.02), whereas patients who participated in the STEVIE trial had a significantly increased probability of achieving a complete response compared to daily practice patients (HR 10.08, *p* < 0.01) (Table 3). The main reasons for treatment discontinuation were toxicity (*n* = 22) and tumour progression (*n* = 15). Retreatment with vismodegib (*n* = 12) led to a response in eight patients, six of them eventually developed progressive disease again. Six patients died due to the laBCC.

**Table 3:** HR with 95% CI for complete response in locally advanced basal cell carcinoma associated with patient and tumour characteristics (*n* = 44).

Characteristic	HR with 95% CI univariable analysis	*P* value	HR with 95% CI multivariable analysis	*P* value
Age (per year)^a^	0.99 (0.96–1.03)	0.85		
Sex				
Male	1.00			
Female	1.78 (0.63–5.07)	0.28		
Tumour size (per cm)^b^	0.91 (0.79–1.06)	0.24	0.77 (0.62–0.95)	*0.02*
Tumour location				
Not on the head	1.00	0.86		
On the head	0.90 (0.25–3.18)			
Tumour subtype				
Non-infiltrative	1.00	0.16	1.00	0.06
Infiltrative	0.46 (0.16–1.35)		0.21 (0.04–1.08)	
Bone invasion				
No	1.00	0.67		
Yes	0.78 (0.25–2.46)			
Previous therapy				
No	1.00	0.44	1.00	0.22
Yes	0.44 (0.24–1.86)		0.46 (0.13–1.58)	
Previous radiotherapy				
No	1.00	0.61		
Yes	1.40 (0.39–5.06)			
Participant in trial				
No	1.00	0.09	1.00	*<0.01*
Yes	2.38 (0.86–6.58)		10.08 (2.14–47.43)	

HRs for complete response with 95% CI in patients with laBCC.

HR > 1 and HR < 1 indicate increased and decreased probability of response, respectively, where categories with HR = 1 were used as the reference category. *P* < 0.05 is considered statistically significant.

*HRs* hazard ratios, *95% CI* 95% confidence interval.

^a^The HR for age represents the increase in probability per year.

^b^The HR for tumour size represents the increase in probability per cm.

### MBCC

Eleven patients received vismodegib for metastasised BCC; none of them had BCNS (Fig. 1). One patient had been treated for the primary laBCC with vismodegib and surgery 4.6 years before. Primary tumours were located in the head and neck region in 46% of patients (*n* = 5), on the trunk in 36% (*n* = 4) and on the extremities in 18% (*n* = 2). The sites of metastases were: regional lymph nodes 27% (*n* = 3), distant lymph nodes 9% (*n* = 1), lungs 55% (*n* = 6) and bones 18% (*n* = 2). Median self-reported tumour presence was 5 years (range, 0.3–22 years) and size was 14.5 cm in diameter (range, 4.0–22.0 cm). All tumours with known subtype (*n* = 7) were infiltrative. At the start of treatment, bone invasion was present in 55% of the patients (*n* = 6). Of all mBCC patients, four did not receive any previous therapy and six had received previous surgery for the primary BCC (Fig. 1c). The median age at the start of treatment was 70 years (range, 52–81 years).

#### Effectiveness

Of the 11 mBCC patients, one had previously been treated for the mBCC with vismodegib abroad, leaving ten patients for the effectiveness analysis. Median PFS was 11.7 months (95% CI, 5.2–17.5). At 3 months after the start of vismodegib, the probability of partial response was 52.0% (95% CI, 25.5–83.9).

The main reason for treatment discontinuation was tumour progression (*n* = 6). Only one patient achieved a complete response, which currently lasts for >2 years without treatment. This patient only had a regional lymph node metastasis and received previous surgical treatment of the primary BCC. After progressive disease, two patients were treated with radiotherapy, one with surgery, two with anti-programme death-1 inhibitors, two are not treated yet, and three patients died.

### Multiple BCCs in BCNS

Nineteen BCNS patients received vismodegib for multiple BCCs. At the start of vismodegib treatment, the median age was 46 years (35–71 years). One patient had previously been treated with vismodegib for this indication abroad and two patients received vismodegib previously for a laBCC, leaving 16 patients for the effectiveness analysis. Median PFS was 19.1 months (95% CI, 7.4–20.2). Numbers were too small to compare effectiveness in clinical trial and daily practice patients. In one patient, the time of response was unknown. In the remaining 15 patients, the probability of achieving partial response within 3 months after the start of vismodegib was 93.3% (95% CI, 74.0–99.6) and probability of complete response after 6 months of treatment was 40.8% (95% CI, 19.3–72.2). The main reason for treatment discontinuation was toxicity (*n* = 13).

Twelve patients (63%) received ≥2 treatment sequences, with a maximum of four sequences (Fig. 1). The median treatment break duration was 11.2 months (range 2.2–54.2 months). All patients responded to vismodegib in all the following sequences.

### Multiple BCCs in non-BCNS patients

Notably, five non-BCNS patients received vismodegib for multiple BCCs: three xeroderma pigmentosum patients and two HF-BCC patients (Fig. 1). Numbers were too small to perform effectiveness analyses. Reasons for termination of treatment were toxicity (*n* = 2) and end of trial (*n* = 2). One HF-BCC patient has been treated successfully alternating 3 months on and off vismodegib 150 mg daily for >3 years.

### Safety analysis

In total, 409 TEAEs were noted in all sequences (Table 4). Of those TEAEs, 77% were grade 1 or 2, 2.5% were grade 3 and only 1 patient experienced a grade 4 TEAE (liver toxicity); for the other TEAEs, the grade was not mentioned in the medical file. All patients experienced at least one TEAE, with a median number of four TEAEs per patient (range, 1–12 TEAEs) in the first treatment sequence. Patients who restarted treatment experienced the same TEAEs as in the previous sequence. Of all the side effects, 42% resolved, 19% was still present at the last control and for 39% this information was not noted in the patient file.

**Table 4:** TEAEs per treatment sequence.

TEAEs, *n*	Sequence 1, *n* = 78^a^	Sequence 2, *n* = 22^a^	Sequence 3, *n* = 8	Sequence 4, *n* = 5	Resolved	Not resolved	Not reported
Muscle spasms, 81	58 (74%)	14 (64%)	7 (88%)	2 (40%)	35 (43%)	12 (15%)	34 (42%)
Dysgeusia, 76	56 (72%)	14 (64%)	4 (50%)	2 (40%)	35 (46%)	13 (17%)	28 (37%)
Alopecia, 55	47 (60%)	6 (27%)	2 (25%)	–	24 (44%)	9 (16%)	22 (40%)
Weight loss, 29	22 (28%)	5 (23%)	2 (25%)	–	7 (24%)	5 (17%)	17 (59%)
Fatigue, 21	19 (24%)	1 (5%)	1 (13%)	–	4 (19%)	9 (43%)	8 (38%)
Decreased appetite, 17	12 (15%)	4 (18%)	1 (13%)	–	8 (47%)	6 (35%)	3 (18%)
Diarrhoea, 15	11 (14%)	2 (9%)	1 (13%)	1 (20%)	6 (40%)	1 (7%)	8 (53%)
Nausea, 13	9 (12%)	3 (14%)	1 (13%)	–	6 (46%)	1 (8%)	6 (46%)
Headache, 9	9 (12%)	–	–	–	–	–	–
Myalgia, 8	7 (9%)	1 (5%)	–	–	–	–	–
Hepatotoxicity, 6	4 (5%)	2 (9%)	–	–	–	–	–
Dizziness, 6	5 (6%)	1 (5%)	–	–	–	–	–
Abdominal pain, 4	4 (5%)	–	–	–	–	–	–
Ageusia, 4	4 (5%)	–	–	–	1 (25%)	–	3 (75%)
Asthenia, 2	2 (3%)	–	–	–	–	–	2 (100%)

*TEAE* treatment-emergent adverse event.

^a^All individual patients who received the first or second treatment sequence in the Netherlands.

## Discussion

In this retrospective cohort study, data were provided about vismodegib use in the Netherlands. In the national guidelines, the indication for vismodegib treatment is “reserved only for patients with an aBCC where surgery and radiotherapy are ineffective or encounter major objections”. In a population of ~17.2 million people and a suspected incidence of BCC of 3–10% per year, only 80 patients have been treated with vismodegib in a period of almost 8 years.^10,11^ Over one-third of these 80 patients were initially included in a clinical trial, which indicates the reluctance to prescribe vismodegib in the Netherlands.

Unique for our study is the reflection of all data concerning the use and effectiveness of vismodegib and the course of treatment after vismodegib discontinuation. We found a median PFS of 10.3 months for the indicated laBCC, 11.7 months for mBCC, and 19.1 months for BCNS. Comparable results for the aBCC population were found in other studies. The ERIVANCE trial found a median PFS of 12.9 months for the laBCC group and 9.3 months for the mBCC group, and the STEVIE trial found 13.2 months for the mBCC group.^6,12^ However, there was one exception, the STEVIE trial found a much longer PFS of 23.2 months in the laBCC group. The long duration of PFS in the laBCCs of the STEVIE trial is remarkable. An explanation might be a difference in included tumour types between our country and the STEVIE trial. Information on the subtype and size of BCCs included in the STEVIE trial is not available. In our country, vismodegib was exclusively prescribed after evaluation of the tumour in a multidisciplinary tumour board, including a head and neck surgeon, a radiotherapist and an oncologist, which may result in defining a tumour “irresectable and not suitable for radiotherapy” at a more advanced stage. It can be speculated that tumours with a more advanced nature do worse and will show progression at an earlier stage. This hypothesis is confirmed by analyses of our own data in which we found that larger tumours have a lower probability of complete response versus smaller tumours. A second explanation for the difference in PFS between our study and the STEVIE can be the retrospective nature of our study in which effectiveness outcomes relied on the accuracy of record keeping and the frequency of patient visits. Less meticulous measurements in daily practice might affect the assessed PFS. Finally, the definition of tumour progression differed between the studies: in the STEVIE trial, it was defined as >20% increase in size, taking as reference the smallest tumour size measured during the study, whereas in our study, progression as noted by the physician was additionally defined as disease progression. In the latter definition of progression, the increase could be <20%, but with more other complaints, such as bleeding, pain or ulceration. This could have led to a shorter PFS in our study.

A few patients achieved a prolonged complete response, a phenomenon that has previously been described in a French population.^13^ To determine what tumour types achieved a complete response, we compared several factors for probability of complete response in the multivariable Cox regression analysis (tumour size, histologic subtype, previous treatment and clinical trial participation). Irrespective of the other variables, patients with laBCCs that participated in the STEVIE trial had a very high probability of achieving a complete response compared to patients treated in daily practice. This higher effectiveness of treatments in patients participating in randomised controlled trials is known as the Hawthorne effect.^14^

According to the FDA (United States Food and Drug Administration) and EMA (European Medicines Agency) guidelines, vismodegib is only approved for the treatment of aBCC. Data on effectiveness for other indications are sparse and no such data are expected in the near future as there are currently no such clinical trials registered. In our cohort, 22 patients (26%) received vismodegib for a multiple BCC indication (20% BCNS, 4% XP and 2% HF-BCC patients). The large number of BCCs places a heavy burden on these patients and a therapy that can treat all lesions at once is very desirable.^15^ In line with previous clinical trials, we found a high effectiveness of vismodegib in this patient population, but the majority of patients discontinued due to side effects. The frequency of most side effects was somewhat lower than in the STEVIE and ERIVANCE trials.^6,16^ A possible explanation is the retrospective nature of our study. Also, the shorter treatment duration could be causative, as it was found in the STEVIE trial that the frequency of most side effects increased with the treatment duration.^6^ Two differences in side effects compared to previously published trials are notable: (1) a very low frequency of weight loss (28 vs. 41%) and (2) a higher frequency of dysgeusia (72 vs. 55%).^6^ Weight measurement was obligatory in the STEVIE trial, but sometimes omitted in real life, which can explain the difference in the frequency of weight loss. We cannot explain the higher frequency of dysgeusia. However, we hypothesise that its inconvenience stresses patients more to mention this at their consultation, even if not specifically asked for, whereas in the STEVIE trial, all side effects had to be checked systematically.

To allow patients to recover from side effects, different intermittent dosing schedules were used. In the two intermittent vismodegib dosing regimens of the MIKIE trial (vismodegib daily alternate with 8 weeks of placebo), side effects still appeared substantial.^8^ From our data, it becomes clear that in daily practice patients often have a much longer treatment break. Although our data show a lower frequency of side effects in the following sequences, it does not mean patients will endure less side effects in the following sequence. As most patients stopped treatment due to side effects, selection of patients who have experienced less severe side effects could have occurred in the group that was treated with a second sequence. Moreover, the median treatment durations of the following sequences were shorter compared to the first sequence (6.4 months in the first, 5.3 months in the second, 3.3 in the third and 4.8 in the fourth sequence). From the STEVIE trial, it is known that the median time to onset of alopecia is 5.6 and dysgeusia is 6.5 months, which might explain why those side effects were reported less in the second sequence.^7^ Lastly, ~20–30% of the patients in the following sequence received an alternate dose of vismodegib, specifically to lower side effects.

Seven BCNS patients have already been treated successfully for ≥3 times in 8 years and one HF-BCC patient is treated successfully for years with 3 months on and off vismodegib treatment. Unfortunately, there is currently no information on the effects of lifelong intermittent treatment on the general health of patients and on the progression of BCC size and aggressiveness during treatment breaks. Although it is likely that intermittent vismodegib and multiple surgical procedures both affect the quality of life in this patient group, it is currently unknown which strategy has the least impact. Clustering data from different BCNS centres worldwide can provide the best answers to these questions.

This study provides important information on vismodegib effectiveness and the course of treatment after vismodegib discontinuation. Median PFS was less than a year for aBCCs. Future research should focus on treatment combinations or options after vismodegib failure and defining which patients can achieve a prolonged complete response. In BCNS patients, PFS is longer than in aBCCs, but treatment is often discontinued due to side effects. Retreatment remains effective and can be applied in various schedules.

## Supplementary information

Ethics committees

## Data Availability

Data are not available in a public database yet, but authors are willing to share data.
