# Supplementary material for: Eight years of experience with vismodegib for advanced and multiple basal cell carcinoma patients in the Netherlands: a retrospective cohort study
Source: Br J Cancer. 2021 Jan 19;124(7):1199–206. doi: 10.1038/s41416-020-01220-w (PMC8007568; doi:10.1038/s41416-020-01220-w)
Supplement: Supplementary file 1 — Ethics committees [file 41416_2020_1220_MOESM1_ESM.docx]

Medical ethical committee’s:

- Medisch-ethische toetsingscommissie azM/UM
  P. Debyelaan 25
  Postbus 5800
  6202 AZ Maastricht
- Medisch Ethische Toetsings Commissie Erasmus MC
  Postbus 2040
  3000 CA Rotterdam
  Kamer Ae-337
- Medische Ethische Toetsings Commissie Leiden-Den Haag-Delft
  postzone P5-P
  Postbus 9600
  2300 RC Leiden
- Medische Ethische Toetsingscommissie AMC
  Kamer TK0-270
  Meibergdreef 9
  1105 AZ Amsterdam
- Medische Ethische Toetsingscommissie UMC Groningen
  Postbus 30 001
  9700 RB GRONINGEN
  HPC: LA15
- Medische Ethische Toetsingscommissie Utrecht
  Huispostnummer D.01.343
  Postbus 85500
  3508 GA UTRECHT
